# Endoscopic findings in periapical surgery. A cross-sectional study of 206 roots

**DOI:** 10.4317/medoral.25311

**Published:** 2022-06-05

**Authors:** Pablo Glera-Suárez, Antonio Pallarés-Serrano, David Soto-Peñaloza, Beatriz Tarazona-Álvarez, Miguel Peñarrocha-Diago, David Peñarrocha-Oltra

**Affiliations:** 1 DDS, MS. Master in Oral Surgery and Implant Dentistry, Department of Stomatology, University of Valencia Medical and Dental School, Valencia, Spain; 2 DDS, MS, PhD. Master in Oral Surgery and Implant Dentistry, Department of Stomatology, University of Valencia Medical and Dental School, Valencia, Spain; 3 DDS, MS, PhD. Assistant Professor of Orthodontics, University of Valencia Medical and Dental School, Valencia, Spain; 4 MD, DDS, MS, PhD. Professor and Chairman, Oral Surgery Unit, Department of Stomatology, Faculty of Medicine and Dentistry, University of Valencia, Spain; 5 DDS, MS, PhD. Full Professor, Oral Surgery Unit, Department of Stomatology, University of Valencia Medical and Dental School, Valencia, Spain

## Abstract

**Background:**

A study is made of the findings of high-magnification rigid endoscopy at the root end surface following apicoectomy of teeth subjected to periapical surgery.

**Material and Methods:**

A cross-sectional study was made of patients subjected to periapical surgery at the Unit of Oral Surgery and Implantology (University of Valencia, Valencia, Spain) between 2011 and 2019. Following apicoectomy, the root end surfaces were inspected, with the evaluation of untreated canals, isthmuses, craze lines, crack lines, opaque dentin and gaps. Likewise, an analysis was made of the association between patient age and the tooth type and restoration and the presence of craze lines, cracks, opaque dentin and gaps.

**Results:**

The final sample consisted of 168 patients subjected to periapical surgery, with 177 operated teeth and 206 roots. Untreated canals were observed in 14 roots (6.8%). Isthmuses were identified in 74 roots (35.9%), particularly in the mesial root of the lower first molar (94.1%). In turn, craze lines were identified in 8.3% of the roots, cracks in 3.9%, and gaps in 53.4%. The prevalence of opaque dentin was 78.3%, with a greater presence in posterior teeth (90.3% in premolars and 86.2% in molars) than in anterior teeth (50.6%) (*p*<0.001). Patient age and tooth restoration showed no correlation to the studied parameters.

**Conclusions:**

Craze lines and crack lines were observed in less than 10% of the roots, though opaque dentin was identified in 73% of the roots, particularly in posterior teeth, and gaps were found in over half of the canals.

** Key words:**Endoscope, cracks, gaps, opaque dentin, periapical surgery.

## Introduction

Periapical surgery is used to treat persistent chronic apical periodontitis in cases where healing is not achieved (1). Periapical microsurgery has been shown to improve the prognosis, thanks to magnification and illumination of the surgical field with a microscope or endoscope, allowing the detection of features that are not visible to the naked eye (2). Use is also made of instruments such as ultrasonic tips to prepare the retrograde cavity, together with more biocompatible materials such as mineral trioxide aggregate (MTA) (3,4).

The rigid endoscope is a useful tool in periapical microsurgery, being versatile, rapid and convenient to use. It offers simple focusing and zoom functions, easy mobility, and good visibility around the roots (5). The success rate of periapical microsurgery with an endoscope is similar to that obtained with a microscope, though the learning curve is easier with the former instrument (6). With regard to the degree of magnification, medium settings (x8-14) have been proposed for hemostasis, the removal of granulation tissue, the detection and location of roots, apical resection, and preparation and filling of the retrograde cavity. High magnification (x14-26) in turn is used for inspection of the root end surface, retrograde cavity and retrograde filling, with the purpose of detecting untreated canals, isthmuses or microfractures (7,8).

The first study on the root end surface with magnification in periapical surgery was published in 2003 by Slaton *et al*. (9). These authors conducted an *in vitro* evaluation of 50 maxillary teeth in which dentinal cracks were induced for subsequent analysis with a microscope, endoscope and magnification loupe. They described the presence of opaque zones in the apical dentin (“opaque or frosted dentin”), which were associated with tension zones (9). The authors also endorsed the endoscope as the best magnification system for detecting these dentinal cracks, compared with the microscope and loupes.

Von Arx *et al*. subsequently carried out two clinical studies involving analysis of the endoscopic images of the root end surface following apical resection (10,11). In both studies, the most frequent finding in the roots was opaque dentin (79.8-84.1%), particularly in premolars and molars. They also reported a prevalence of gaps of 49.3% (11) to 83.3% (10), and a very low presence of craze lines (6.5%) and cracks (3.5-10.1%).

Since only two clinical studies (10,11) have analyzed endoscopic images in periapical surgery to date, and both moreover have been published by the same research group, we decided to carry out the present study, analyzing high-magnification endoscopic images of the root end surface following apicoectomy in order to assess details not detecTable without the use of magnification measures, such as untreated canals, isthmuses, craze lines, crack lines, opaque dentin and gaps.

## Material and Methods

- Study design

A cross-sectional study was conducted at the Oral Surgery and Implantology Unit (Department of Stomatology, University of Valencia Medical and Dental School, Valencia, Spain) in patients subjected to periapical surgery between September 2011 and December 2019. The study was conducted in abidance with the Declaration of Helsinki (1975 as revised in 2013) regarding biomedical research in human subjects, and was approved by the Ethics Committee of the University of Valencia (Protocol ref.: 1126870).

The present manuscript is reported according to the Strengthening the Reporting of Observational Studies in Epidemiology (STROBE) statement for cohort studies (www.strobe-statement.org).

- Sample selection

The following inclusion criterion was established: periapical surgeries performed using a rigid endoscope to obtain high-magnification intraoperative photographs. The exclusion criteria were: roots presenting vertical fractures implying extraction of the tooth, and poor quality endoscopic images or images with artifacts precluding evaluation of the study variables.

- Surgical technique

In all cases local infiltration anesthesia was provided with 4% articaine and epinephrine (1:100,000) (Inibsa®; Llica of Vall, Barcelona, Spain), and all surgeries were performed using a dental operating microscope (Möller® Dental 300, Wedel, Germany). Paramarginal or submarginal incisions were performed. After mucoperiosteal flap release, an ostectomy was carried out with a 1:1 handpiece (W&H®, Bürmoos, Austria) under irrigation with sterile saline solution. Hemostasis was secured with Expasyl™ (Pierre Rolland, Merignac, France).

The apical portion was resected 3 mm, and the root end surface was inspected with a rigid endoscope with 30° forward view and 2.7 mm in diameter (HOPKINS® optics model 7207 BA, Karl Storz-Endoskope®, Tuttlingen, Germany). Images were captured and processed using a documentation device providing 5600 K daylight coloration, with a 50 W (1000 lumens) halogen lamp illumination source (TELE PACK™ PAL Control Unit 200430-20, Karl Storz-Endoskope®) and using a digital camera with Parfocal Zoom Lens, f=25-50 mm (2x) (TELECAM® PAL color system, Karl Storz-Endoskope®). Methylene blue dye was used in cases in which a crack or a root fracture was suspected, for confirmatory purposes. The retrograde cavities were then prepared 3 mm in depth with ultrasonic retrotips (Piezomed®, W&H, Bürmoos, Austria), followed by retrofilling with mineral trioxide aggregate (MTA) (Dentsply®, Tulsa Dental Specialties, Tulsa, OK, USA). Intraoperative photographs were obtained using the rigid endoscope with the highest possible magnification. Tension-free flap closure was performed using 6/0 suture material (Polinyl®, Sweden & Martina, Carrare, Italy). The surgical technique has been further detailed in previous publications (12).

- Data collection

Clinical data were compiled, together with radiological studies and endoscopic images of the root end surface following apicoectomy of the patients included in the study. The filed endoscopic images (Karl Storz Telepack PAL 200430 20, Tuttlingen, Germany) were exported and examined on a computer with LED monitor (iMac Pro, Apple, Cupertino, CA, USA) to assess their validity in terms of sharpness and the presence of artifacts (blood, fluid) capable of interfering with evaluation. All the endoscopic images were analyzed by two evaluators (PGS, APS), and disagreement was resolved by consensus with an expert (MPD).

**Figure 1:**
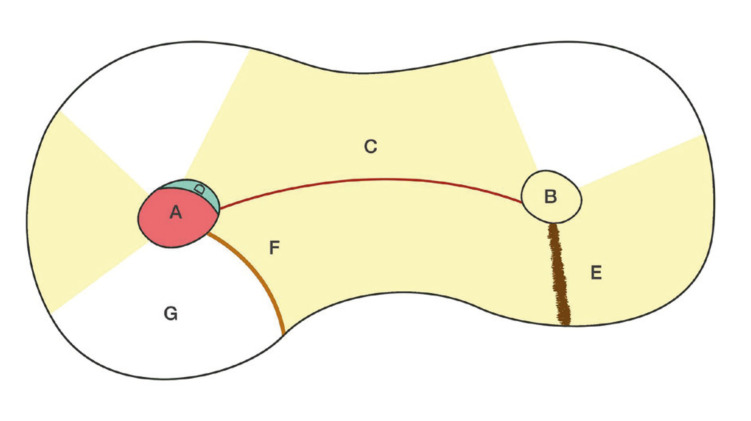
Illustrative view of the endoscopic findings. A: orthograde treated canal; B: untreated canal; C: Isthmus; D: gap, E: crack line; F: craze line; G: opaque dentin.

The primary study variables were the presence of untreated canals (untreated via the orthograde route), isthmuses (joining lines between two or more canals), craze lines (dark lines without loss of root dentin structure) (10), crack lines (fissures in the root dentin), frosted dentin (area of root dentin of a whitish appearance contrasting with the conventional yellow / gray tone of dentin) and gaps (remaining unfilled spaces between the root canal filling material and the dentin wall). In addition, we precisely determined the location of the craze lines, crack lines, opaque dentin and gaps in the root, defining four zones: buccal, lingual, mesial and distal. The primary study variables are depicted in Fig. 1.

The secondary study variables were the age of the patient (< 45 years or > 45 years), the type of treated tooth (anterior teeth: central and lateral incisors, and canines; and posterior teeth: premolars and molars), and the presence of tooth restorations with a post or pin.

- Statistical analysis

A descriptive analysis of the study variables was made. Inferential analyses were performed to explore the association between the prevalence of the different findings, and to establish associations with secondary variables (e.g., type of tooth, patient age, and restoration with pin or post), based on the chi-square test.

The chi-square equality of proportions test was used to establish those findings more likely to appear in certain zones of the root than in others. The level of significance was established as 5%, with statistical significance being considered for *p* < 0.05.

## Results

Out of 182 patients subjected to periapical microsurgery with an endoscope, first endoscopic evaluation of the root end surfaces led to the exclusion of 14 roots due to poor resolution of the images. The final study sample thus consisted of 168 patients, with 177 teeth and a total of 206 roots. There were 93 women (55.4%) and 75 men (44.6%), with an overall mean age of 46.7 ± 13.8 years (range 18-81).

The most frequently treated tooth was the upper lateral incisor (16.4%), followed by the upper central incisor (14.1%) and the upper first molar (12.4%). Of the total treated teeth, 74.8% were located in the maxilla and 25.2% in the mandible. The upper premolars presented one or two roots, while the lower premolars presented a single root in all cases. The most frequently treated root of the posterior teeth was the mesiobuccal root of the upper first molar (8.7%). The distribution of the teeth and roots is shown in Table 1.

**Figure 2:**
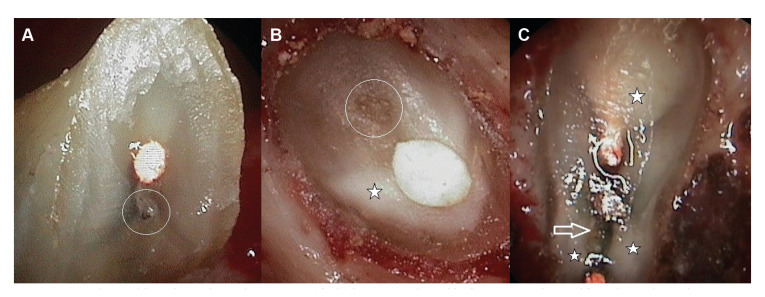
A: Canal not subjected to orthograde treatment (circle) in an upper lateral incisor; B: Canal not subjected to orthograde treatment (circle) in an upper central incisor. Note the presence of opaque dentin (star); C: Joining isthmus between two canals (arrow) in the mesial root of a lower first molar. Zones of opaque dentin are also seen (star).

**Figure 3:**
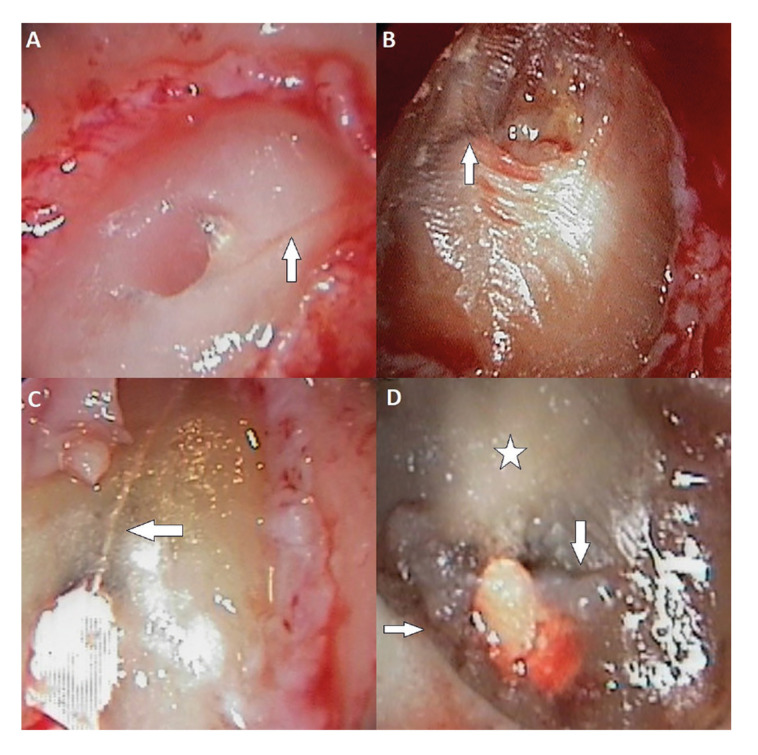
A: Craze line (arrow) in an upper canine; B: Craze line (arrow) in the distal root of an upper first molar; C: Crack lines (arrow) in the mesial root of a lower first molar. Note the presence of opaque dentin (star); D: Crack lines (arrow) in the distal root of an upper first molar. Opaque dentin is also observed (star).

- Primary variables

1) Untreated canals were identified in 14 roots (6.8%). Of these, 5 corresponded to anterior teeth: three upper lateral incisors, one upper central incisor, and one lower central incisor (Fig. 2). Three canals were found in a mesiobuccal root of an upper first molar, and in a mesial root of a lower first molar.

2) Isthmuses were recorded in 74 roots (35.9%), being found in approximately one-half of the molars (47.7%), particularly in the mesiobuccal root of the lower first molar (94.1%) (Fig. 2), and in 21% of the premolars.

3) Craze lines (Fig. 3) were seen in 17 of the 206 roots (8.3%). In two of the 17 roots two craze lines were recorded, while another presented three such lines. Craze lines were more frequent in the mesial (33.3%) and distal zones (33.3%) of the roots than in the buccal (19%) and lingual zones (4.8%).

4) Crack lines (Fig. 3) were detected in 8 of the 206 roots (3.9%). One root presented two cracks and another had three. Dentin cracks were more frequent in the buccal zone of the roots (62.5%), followed by the lingual (25%), distal (25%) and mesial zones (12.5%).

5) Opaque dentin (Fig. 2, Fig. 3) was observed in 152 roots (73.8%). The root zones with the greatest presence of opaque dentin were the mesial zone (30.2%), followed by the buccal (25.4%), lingual (22.8%) and distal zones (21.6%).

**Table 1:**
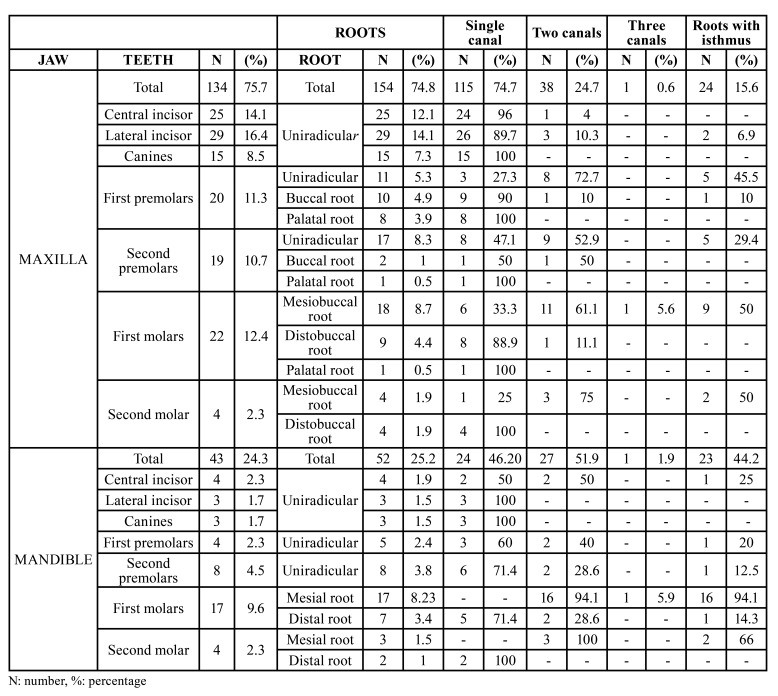
Distribution of teeth (n = 177) and roots (n = 206), and frequency of canals and isthmuses according to groups of roots.

**Figure 4:**
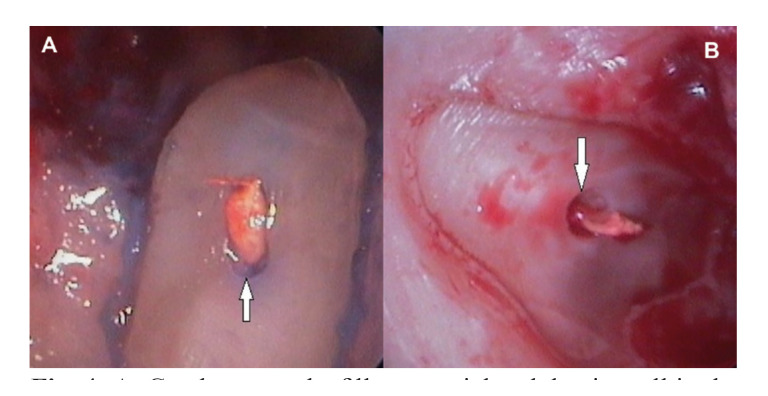
A: Gap between the filler material and dentin wall in the buccal root of an upper first premolar; B: Gap between the filler material and dentin wall in the distal root of an upper first molar.

6) Gaps (Fig. 4) were detected in 110 roots (53.4%), and were slightly more numerous in the buccal (34.5%) and lingual zones of the root (25.4%) than in the mesial (20.9%) and distal zones (19.1%).

The distribution of craze lines, crack lines, opaque dentin and gaps according to the different root zones is described in Table 2.

**Table 2:**
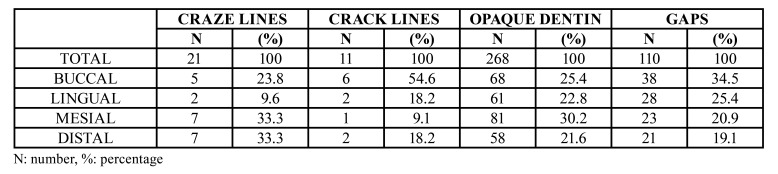
Distribution of craze lines, crack lines, opaque dentin and gaps according to root zone (buccal, lingual, mesial or distal).

- Association to secondary variables

1) Patient age: A total of 148 roots (71.8%) belonged to the group of patients aged 45 years or older. No statistically significant differences were observed on relating the presence of craze lines (*p* = 0.07), cracks (*p* = 0.315), opaque dentin (*p* = 0.325) or gaps (*p* = 0.529) to the two age groups (Table 3).

2) Type of tooth: The presence of opaque dentin was significantly greater in posterior teeth (90.3% in premolars and 86.2% in molars) than in anterior teeth (50.6%) (*p* < 0.001). The presence of craze lines (*p* = 0.652) and crack lines (*p* = 0.157) was slightly greater in posterior teeth, though statistically significant differences were not recorded. There were no differences in the presence of gaps according to the type of tooth (Table 3).

3) Tooth restored with or without pin or post: No statistically significant differences were observed in relation to the presence of craze lines (*p* = 0.09), crack lines (*p* = 0.105), opaque dentin (*p* = 0.375) or gaps (*p* = 0.425) (Table 3).

**Table 3:**
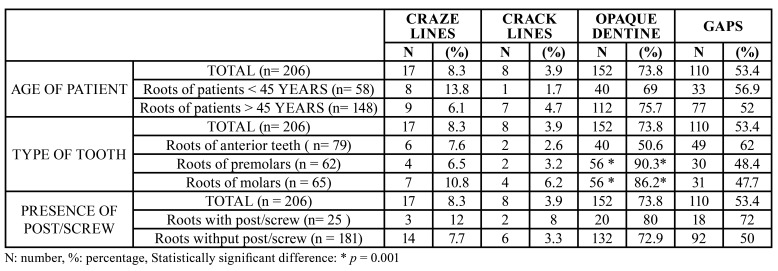
Presence of craze lines, crack lines, opaque dentin and gaps according to secondary variables.

## Discussion

The aim of the present study was to inspect root end surfaces following apical resection using a rigid endoscope in order to allow the detection of features that are not visible to the naked eye, and to associate them to the age of the patient, the type of tooth and the presence of posts or pins in the treated teeth.

In recent years, one of the main advances in periapical surgery has been the introduction of magnification and illumination systems such as the endoscope, microscope, or magnification loupe (13-16). Microscopes and loupes afford the magnification and illumination needed for the operation, though there has been growing interest in the use of endoscopes as an alternative for guaranteeing adequate magnification and illumination in periapical surgery (15,17), since the learning curve is easier and endoscopy has shown great precision in diagnosing microstructures in periapical surgery (13,17,18).

Two of the main causes of conventional endodontic treatment failure are the presence of unfilled root canals and joining isthmuses between canals. In the present study, the prevalence of isthmuses was 47.7% in molars, with particular involvement of the mesial root of the lower first molar (91.1%). These data are consistent with the observations of previous studies reporting a high frequency of isthmuses in the mesial root of the lower first molar (10,19,20). The presence of isthmuses has been shown to significantly increase the likelihood of periapical surgery failure (21); correct diagnosis and preparation of the retrograde cavity in roots with isthmuses is therefore crucial in order to ensure predicTable treatment.

With regard to craze lines and crack lines, it has been seen that defects of this kind (22) may be caused both by orthograde canal treatment (23) and by retrograde cavity preparation during periapical surgery (24). To date, only three periapical surgery studies have intraoperatively evaluated the possible causes of conventional endodontic treatment failure (10,11,25). In 2011, Song *et al*. recorded a 1.2% prevalence of dentin cracks in the roots inspected with a microscope (25). That same year, von Arx *et al*. (10), using magnification with a rigid endoscope, reported a prevalence of craze lines and crack lines of 6.5% and 3%, respectively - most of these defects being located in the buccal section of the root. In 2017, von Arx *et al*. (11) published another clinical study in which endoscopy identified crack lines in 10% of the roots. In the present study, craze lines were detected in 8.3% of the cases, while the prevalence of crack lines was 3.9%. These data are similar to those published by von Arx *et al*. (10,11). While the impact of crack lines upon the clinical outcomes of endodontic surgery is not fully clear, it has been reported that they can give rise to bacterial colonization (26), can affect sealing of the retrograde cavity (27), and may even progress to vertical fracture (8,22). In concordance with von Arx *et al*. (10), we found no correlation between the age of the patient and the occurrence of crack lines or craze lines, and most of the dentin cracks were moreover located in the palatal zone of the root (62.5%). However, no association was found with the presence of restorations with posts or pins, and the presence of cracks was not seen to be more frequent in premolars than in other types of teeth (10). This lack of an association may be explained by the few teeth restored with posts or pins in the study sample.

Another of the features analyzed in our study was opaque dentin, characterized by a shift in dentin color from yellow-gray to white. This characteristic was identified in 2003 by Slaton *et al*. (9) following apical resection in an *in vitro* study on the formation of dentin cracks. The authors described opaque dentin as appearing in tension zones. In 2006, Paque *et al*. (28) associated the presence of opaque dentin to zones with permeable rather than sclerotic dentin tubules. In 2014, Russel *et al*. (29) studied what they called the butterfly effect - an optical phenomenon seen in cross-sectional visualization of a root after apical resection - and found the density of the tubules to be significantly greater in the buccolingual (opaque) zones than in the mesiodistal (translucent) zones corresponding to the mentioned butterfly effect. In a later study, these same authors evaluated dentin hardness associated with the butterfly effect (30) and found hardness to be significantly greater in the mesial and distal zones (butterfly effect) than in the buccolingual zones. The authors concluded that this could explain the high prevalence of vertical root fractures that occur in the buccolingual direction (26,30). The clinical implications of opaque dentin are therefore still not fully clear, as they might represent precursors of dentin cracks or fractures in these root zones.

Von Arx *et al*. reported the prevalence of opaque dentin to be 79.8% (10) and 84.1% (11), and found such dentin to be more frequent in posterior teeth and in the buccal zone of the root. In the present study, and in concordance with the data published by von Arx *et al*. (10,11), we detected the presence of opaque dentin in 152 roots (73.8%), and found the posterior teeth to be significantly more affected (*p* < 0.001). Nevertheless, the distribution by root zones was quite homogeneous, with no clear differences evidencing a greater presence of opaque dentin in some zones of the root than in others. In turn, no association was found between the presence of posts and pins and the age of the patient.

Lastly, one of the main causes of conventional endodontic treatment failure has been shown to be the presence of a gap between the root canal filling material and the walls of the root canal. We recorded a total of 110 gaps (53.4%), and these were slightly more prevalent in the buccal (34.5%) and lingual zones of the root (25.4%) than in the mesial (20.9%) and distal zones (19.1%). These observations are in line with those published by von Arx *et al*. (10,11), who reported a greater incidence of gaps in the buccal and lingual sections of the roots. In an attempt to explain these observations, the authors speculated that instrumentation and filling of the canals is simpler in the mesial and distal zones than in the buccal and palatine zones, and that the oval shape of the root canals also conditions gap formation.

The present cross-sectional study contributes interesting information on the prevalence of the features that can be diagnosed at the root end surface of the tooth after apical resection using a rigid endoscope as a magnification and illumination system. However, prospective and controlled studies are needed to determine whether there is a long-term association between such parameters and the periapical surgery success rate. Additionally, there are limitations regarding the use of the endoscope, such as fogging or the need to correctly focus the root every time we use the instrument. A possible line for future research could be the study of the long-term healing of those roots presenting opaque dentin, craze lines or crack lines compared with a control group without such defects, with a view to establishing possible influences upon healing probability.

## Conclusions

Craze lines and crack lines were observed in less than 10% of the roots, though opaque dentin was detected in 73% of the roots - particularly in posterior teeth - and gaps were moreover found in over half of the canals. The age of the patient, and restorations with posts or pins, had no influence upon the endoscopic findings.
